# *In vitro* and *in vivo* Evaluation of Synergism between Anti-Tubercular Spectinamides and Non-Classical Tuberculosis Antibiotics

**DOI:** 10.1038/srep13985

**Published:** 2015-09-14

**Authors:** David F. Bruhn, Michael S. Scherman, Jiuyu Liu, Dimitri Scherbakov, Bernd Meibohm, Erik C. Böttger, Anne J. Lenaerts, Richard E. Lee

**Affiliations:** 1 Department of Chemical Biology and Therapeutics, St. Jude Children’s Research Hospital, Memphis, Tennessee, USA; 2 Mycobacterial Research Laboratories, Department of Microbiology, Colorado State University, Fort Collins, Colorado, USA; 3 Institut für Medizinische Mikrobiologie, Nationales Zentrum für Mykobakterien, Universität Zürich, Zürich, Switzerland; 4 Department of Pharmaceutical Sciences, College of Pharmacy, University of Tennessee Health Science Center, Memphis, Tennessee, USA

## Abstract

Spectinamides are new semi-synthetic spectinomycin derivatives with potent anti-tubercular activity. The reported synergism of the precursor spectinomycin with other antibiotics prompted us to examine whether spectinamides sensitize *M. tuberculosis* to other antibiotics not traditionally used in the treatment of tuberculosis to potentially expand therapeutic options for MDR/XDR Tuberculosis. Whole cell synergy checkerboard screens were performed using the laboratory strain *M. tuberculosis* H37Rv, lead spectinamide 1599, and a broad panel of 27 antibiotics. *In vitro*, 1599 synergized with 11 drugs from 6 antibiotic classes. The observed synergy was tested against clinical isolates confirming synergy with Clarithromycin, Doxycycline and Clindamycin, combinations of which were taken forward for *in vivo* efficacy determination. Co-administration of 1599 and clarithromycin provided additional bacterial killing in a mouse model of acute tuberculosis infection, but not in a chronic infection model. Further studies indicated that mismatched drug exposure profiles likely permitted induction of phenotypic clarithromycin resistance and subsequent loss of synergism. These studies highlight the importance of validating *in vitro* synergism and the challenge of matching drug exposures to obtain a synergistic outcome *in vivo*. Results from this study indicate that a 1599 clarithromycin combination is potentially viable, providing the drug exposures can be carefully monitored.

More than 130 years since after its identification as the causative agent of tuberculosis in 1882, *Mycobacterium tuberculosis* remains a severe global health threat, has infected more than one third of the world’s population, and is responsible for almost 2 million deaths annually^1^. Although most common in the developing world, more than 10,000 cases of tuberculosis and >500 associated deaths occur in the United States annually^2^. Adept at evasion of the human host’s immune system, *M. tuberculosis* establishes persistent infections and forms granulomas in the lung^3^. There the bacteria can remain in a latent state for decades before reactivating and disseminating to cause an active disease state^4^. The complexity and challenge of killing granuloma-resident bacilli is increasingly recognized as the result of multiple subpopulations, even within a single granuloma, each with distinct drug-susceptibility and resistance profiles^5^,^6^.

Treatment of *Mycobacterium tuberculosis* infections is lengthy and hindered by the emergence of drug resistance^7^. Standard treatment of drug-susceptible infections requires a 2 month initial phase with daily administration of frontline drugs rifampin, isoniazid, pyrazinamide, and ethambutol followed by a 4 month continuation phase composed of isoniazid and rifampin. Patient adherence to this lengthy regime is challenged by antibiotic-associated toxicity and inadequate access to individual regime components, particularly isoniazid for which supply shortages are not uncommon^8^. Underexposure of drug is proposed to permit selection of genetic mutants resistant to frontline drugs and further exacerbates the clinical challenge of managing tuberculosis by fueling the development of acquired drug resistance^9^. Drug resistant tuberculosis isolates can be spread from person to person, often with low fitness cost for the bacteria, and radically increases the length and cost of treatment for the disease^10^,^11^.

In response to the increasing prevalence of multidrug-resistant (MDR) and extensively drug-resistant (XDR) tuberculosis infections, numerous efforts are being undertaken to thwart the spread of this global killer. Several novel scaffolds with unique mechanisms of action (and thus potential to inhibit drug-resistant infections) are in various stages of pre-clinical and clinical development. This includes nitroaromatic prodrugs (PA-824, TBA-354, and delamanid), cell wall active compounds such as SQ109, the structurally unique aminoglycoside apramycin, and the recently approved bedaquiline (TMC207)^12^,^13^,^14^,^15^,^16^,^17^,^18^. Efforts to re-sensitize drug-resistant infections to frontline drugs by adjuvant therapy with non-tubercular agents including efflux inhibitors verapamil and thioridazine have also shown promise and are being pursued^19^,^20^. Synthetic modification of natural products has led to the successful development of treatments for Gram-positive bacterial infections and we have recently applied this approach in the discovery and preclinical development of anti-tuberculars known as spectinamides^21^.

Spectinamides are semi-synthetic derivatives of spectinomycin, an aminocyclitol that binds to a site within the bacterial 30S ribosome (helix 34 of 16S rRNA) distinct from that of other protein synthesis inhibitors, including aminoglycosides and macrolides^22^. Unlike aminoglycosides, spectinomycin does not inhibit human mitochondrial translation, a side effect of aminoglycosides that leads to ototoxicity^23^,^24^,^25^. Despite its potent activity against bacterial ribosomes, spectinomycin is only weakly antibacterial owing to limited intracellular accumulation and resultant access to the ribosomal target. Efflux pump Rv1258c is upregulated in MDR/XDR isolates *in vitro*, implicated in drug tolerance, upregulated in human sputum samples during the course of tuberculosis treatment, and provides *M. tuberculosis* with intrinsic resistance to spectinomycin^5^,^19^,^21^,^26^,^27^. Semi-synthetic spectinamide analogs avoid efflux by Rv1258c, thereby gaining potency against *M. tuberculosis* both *in vitro* and in *in vivo* models of tuberculosis infection^21^.

Spectinomycin was previously shown to synergize with several classes of drugs *in vitro*^28^. While the poor anti-tuberculosis activity of spectinomycin may restrict this synergism *in vivo*, spectinamides are efficacious *in vivo* and, so, afford the opportunity to further investigate the prospects for sensitization and synergism between spectinomycins and antibiotics lacking intrinsic anti-tuberculosis activity. Therefore, we investigated the interaction of spectinamides with a library of antibiotics not currently used in standard TB therapy.

Hits were confirmed in clinical isolates and mechanisms underlying synergy were explored using both genetic and biochemical approaches. These studies indicated that lead spectinamides display favorable interactions with some FDA approved antibiotics including macrolides *in vitro*. Combination treatment with 1599 and clarithromycin provided additive reduction in bacterial loads in an acute model of tuberculosis infection but this interaction was not seen in a lengthier model of chronic tuberculosis infection, apparently likely due to mismatched exposure profiles as we show here. These studies highlight the challenges of matching *in vitro* synergism *in vivo* due to the impact of differing pharmacokinetic profiles and dosing schedules on *in vivo* success of drug combinations identified *in vitro*.

## Results

### Chemically diverse antibiotics synergize with anti-tuberculosis spectinamide 1599 *in vitro*

The interaction of lead spectinamide 1599 with a library of 27 antibiotics not normally used to treat tuberculosis was examined *in vitro* using checkerboard synergy assays (Table 1). In this well-established technique, the reduction in the minimum treatment concentration required to inhibit growth (MIC) is established for 2 compounds alone and in combination. The mutual reductions in MICs are used to calculate the fractional inhibitory concentration index (FICI), where a FICI ≤ 0.5 indicates synergism. Preliminary screening performed with *Mycobacterium tuberculosis* laboratory strain H37Rv indicated synergy (FICI ≤ 0.5) with 13 partner drugs, including trimethoprim, bacitracin, vancomycin, tetracyclines, macrolides, and closely related lincosamides. Encouragingly, the finding of synergism between clarithromycin and 1599 is similar to previously reported synergism between clarithromycin and the parent drug spectinomycin^28^. Despite synergism with tetracyclines, 1599 did not synergize with the structurally-similar glycylcycline tigecycline (FICI = 0.6)^29^. Indifference (no antagonism) was seen between 1599 and aminoglycosides, beta-lactams, nitroimidazoles, and parent molecule spectinomycin. 1599 neither synergized with nor sensitized cells to spectinomycin, suggesting that it’s avoidance of efflux by pump Rv1258c is not due to inhibition of this pump, as the later would be expected to sensitize TB to spectinomycin.

### Spectinamides synergize with tetracyclines, macrolides, and lincosamides to enhance potency against clinical isolates

Representative hits identified in initial screens were verified in checkerboard synergy assays against the laboratory *M. tuberculosis* Erdman strain and two clinical isolates (Table 2). 1599 synergism with trimethoprim and the cyclic polypeptide bacitracin was restricted to laboratory strain H37Rv, as FICI scores determined for clinical isolates ranged from 0.8 to 2.0, indicating indifference. Synergism of 1599 and vancomycin was not tested in clinical isolates since this drug did not synergize with structurally related spectinamides (below), possibly indicating a non-specific interaction. The synergism of 1599 with clindamycin, clarithromycin, and doxycycline was maintained in clinical isolates. Synergism between clindamycin and 1599 was particularly notable, as clindamycin itself was inactive against all *M. tuberculosis* strains investigated (MIC ≥ 200 μg/mL). Drugs synergistic with spectinamide 1599 were also tested for synergy with structurally related spectinamide compounds 1329 and 1445 and their precursor, spectinomycin (Table 2). Synergy with clarithromycin, clindamycin, doxycycline, and tetracycline was equivalent for all three spectinamides and spectinomycin, indicating the structural requirement for synergy was not introduced by the spectinamide modification to the parent molecule. Synergy with trimethoprim and vancomycin was restricted to compound 1599, however. This observation was not studied further since 1599 interaction with these drugs was not seen in clinical isolates and, therefore, is inappropriate for further advancement.

### Synergism of 1599 and clarithromycin is achieved at relevant concentrations

An important consideration for the clinical potential of re-purposed antibiotics is if the concentration required to elicit the desired response, in this case synergy, is therapeutically achievable. To begin answering this question for the 1599 combinations identified, we examined the MIC of partner antibiotics in the presence and absence of 1599 (Table 3). The presence of sub-inhibitory 1599 (0.6 μg/mL) reduced the MIC of the partner drugs examined by over 85%. For example, 1599 decreased the MIC of clarithromycin from 25 to 0.1 μg/mL. This is well below the maximum plasma concentration (C_max_) of clarithromycin achieved in humans (2.3 μg/mL) receiving a single 200 mg oral dose of this antibiotic^30^. Sub-inhibitory concentrations of 1599 also reduced the MIC of other antibiotics to equal or slightly above the C_max_ parameters achieved in humans with a single oral dose of clindamycin (600 mg dose, 3.5 μg/mL C_max_)^31^, Doxycycline (100 mg dose, 1.7 μg/mL C_max_)^32^, and Tetracycline (300 mg dose, 2.5 μg/mL C_max_)^33^. These data suggest that amongst the 1599 synergism partners identified, clarithromycin may have the greatest potential for synergism with spectinamides *in vivo*.

### Co-administration of 1599 and clarithromycin provides a statistically significant improvement in clearance of pulmonary infection loads in an acute but not chronic mouse model of tuberculosis infection

**Figure 1:**
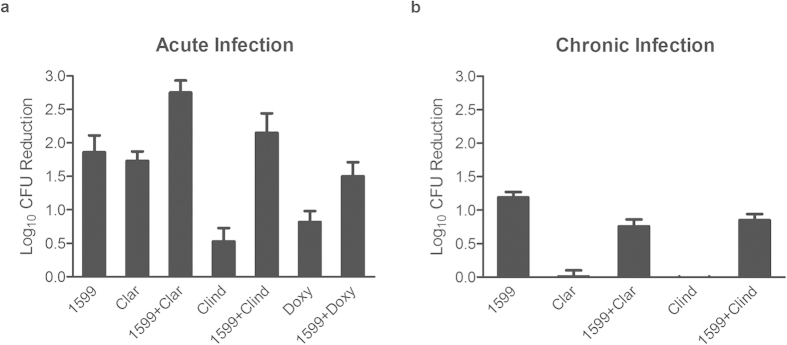
Efficacy of 1599 combinations in acute and chronic infection models. Log_10_ reduction in bacteria in lungs was determined by calculating the difference between bacillary loads in organs from the untreated group and treated groups. Mean log10 CFU reductions per lung ± the standard error of mean (SEM) are presented. (**a**) Murine model of acute tuberculosis infection where low-dose aerosol infected gamma interferon knock-out mice were treated twice a day for 9 consecutive days beginning 14 days post-infection. (**b**) Murine model of chronic tuberculosis infection where low-dose aerosol infected BALB/c mice received treatments once daily for 5/7 days for 28 days beginning 41 days post-infection. Abbreviations: Clar, clarithromycin; Clind, clindamycin; Doxy, doxycycline.

Combinations were tested *in vivo* in a murine model of acute tuberculosis infection. Gamma-interferon knock-out (GKO) mice were infected by low dose aerosol and treatments were initiated 13 days post infection (p.i.) twice per day (BID) for 9 days (Fig. 1a). Monotherapy with 1599 alone reduced bacterial loads in the lungs by 1.9 logs, similar to previous findings^21^. Clarithromycin alone reduced pulmonary loads by 1.7 logs despite having weak activity *in vitro*, which is in agreement with previous findings^34^ and may reflect this antibiotic’s high accumulation within lungs. Co-administration of 1599 with clarithromycin, however, yielded 2.8 logs of killing (Fig. 1a). This improvement in activity compared to monotherapy was statistically significant (p = 0.008 when compared to 1599 group) (Supplementary Table S1). Co-administration of 1599 with clindamycin or doxycyline did not yield a statistically significant difference in pulmonary loads in comparison with 1599 monotherapy (Supplementary Table S1)^21^.

The activity of 1599 (150 mg/kg), clarithromycin (250 mg/kg), and clindamycin (100 mg/kg) were then tested alone and in combination in a mouse model of chronic tuberculosis infection (Fig. 1b). Owing to the protracted nature of the chronic infection trial, it was impractical to dose animals more than once per day. Hence, BALB/c mice were infected by low dose aerosol and treatments were initiated 41 days p.i., once per day (QD), using a 5 of 7 (5/7) day dosing schedule for 30 days^35^,^36^. Compared to carrier treated controls, 1599 reduced bacterial loads by ~1.2 logs (p < 0.001 vs. carrier) in lungs, as we have reported previously (Fig. 1b, Supplementary Table S2). Monotherapy with clarithromycin or clindamycin, however, was completely ineffective at reducing bacterial load in the lungs. Co-administration of 1599 with either clarithromycin or clindamycin failed to increase bacterial clearance as compared to 1599 monotherapy and, in fact, slightly but significantly reduced the activity of 1599 in the lungs.

### Inducible macrolide resistance may restrict efficacy *in vivo* with 5/7 day dosing

**Figure 2:**
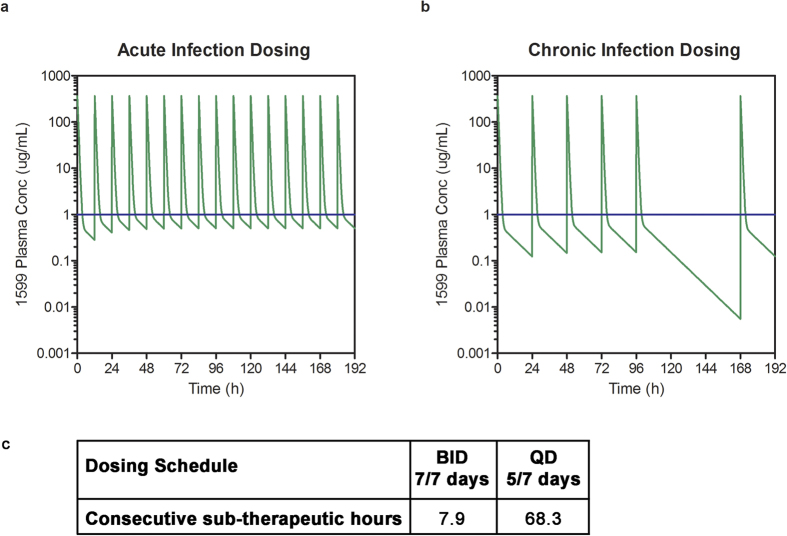
Pharmacokinetic modeling. Free predicted 1599 plasma concentrations during efficacy trials. The MIC for 1599, 1 μg/mL was used to defined the *in vivo* therapeutic concentration (blue line). Predicted plasma concentrations are shown in (**a**) for BID and (**b**) for QD dosing. (**c**) shows the maximum consecutive time of free 1599 concentrations below the MIC for each dosing regimen.

In light of the differing results for the combination of 1599 and clarithromycin in these two distinct infection models, mathematical modeling with the pharmacokinetic parameters of 1599 was performed retrospectively using plasma concentration-time data previously determined in mice after subcutaneous administration of 1599^21^ (Fig. 2). Pharmacokinetic modeling was used to estimate plasma concentrations of 1599 when administered BID or QD to assess if the dosing schedule used *in vivo* may have negatively impacted the efficacy of this combination. The dosing schedule used in the acute infection model (BID dosing for 9 consecutive days) was predicted to result in free plasma concentrations above the MIC of 1599 (defined here as >1 μg/mL) for 7.9 h, representing 34% of the dosing interval. The remaining 66% of the time between doses (7.9 h), free plasma concentrations were predicted to be below MIC. In contrast the dosing schedule used in the chronic infection trial (QD dosing 5/7 days) was predicted to result in periods below MIC of 68.3 h. Additionally, the total daily dose of 1599 and clarithromycin received was decreased by 50% compared to BID dosing when QD dosing was used. This also likely contributed to the lack of efficacy seen in chronic infection trial.

Exposure of mycobacteria to sub-inhibitory concentrations of macrolides (including clarithromycin) induces the bacterium’s *erm* methyltransferase activity that modifies the macrolide binding site to provide antibacterial resistance to this class of drugs. Since 5/7 dosing and lower dosing was predicted to cause periods of monotherapy in which clarithromycin may have induced its own resistance, we next determined if pre-exposure to clarithromycin prevented synergism with 1599. *M. tuberculosis* strains H37Rv and CDC1551 were pre-incubated for 24 hours with sub-inhibitory concentrations of clarithromycin or vehicle prior to performing checkerboard assays (Supplementary Table S3). The combination of 1599 and clarithromycin synergized against untreated H37Rv (FICIs ranged from 0.06 to 0.13), while synergism was greatly ablated in bacteria pre-treated with 0.1 μg/mL clarithromycin (FICIs ranged from 0.65–1.0). Similar results were seen in separate experiments performed with strain CDC1551 (Supplementary Table S4). In the absence of clarithromycin pre-treatment, the lowest concentration of clarithromycin required to reduce the MIC of 1599 ranged from 0.1 to 0.8 μg/mL, which is lower than clarithromycin’s maximum plasma concentration (3 μg/mL) predicted in mice dosed orally at 200 mg/kg clarithromycin^37^. Once bacteria were exposed to monotreatment with clarithromycin (meant to simulate the 2 days off in a 5/7 dosing schedule), the concentration of clarithromycin required to reduce the MIC of 1599 was >50 μg/mL in all experiments. This grossly exceeds the 3 μg/mL peak plasma concentration of this antibiotic that is achievable *in vivo*. These data, combined with pharmacokinetic modeling, suggest that periods of clarithromycin monotherapy resulting from mismatched exposure profiles restricted the synergism *in vivo* by induction of macrolide resistance.

### 1599 potentiates the potency of clarithromycin protein synthesis inhibition

To determine if the strong synergism seen between 1599 and clarithromycin in whole cell assays stemmed from interactions at the ribosomal target, *in vitro* protein translation assays were performed using purified mycobacterial ribosomes. As comparators we included clindamycin and tetracyline, the two other ribosomal inhibitors shown to exhibit synergistic interaction with 1599 in whole cell assays. Titrations of 1599 and partner drugs were prepared (6 concentrations per compound) and the percent inhibition resulting from each of the 36 unique drug concentration reactions measured (Supplementary Fig. S1). A mutual reduction on ribosomal IC_50_ was seen for the combination of 1599 and clarithromycin, where the IC_50_ of clarithromycin was reduced from 1 to 0.2 μg/mL in the presence of sub-IC_50_ 1599. Although titration translation assays did indicate potentiation of protein translation inhibition, the magnitude of this interaction did not match the strong synergism seen in whole cell growth assays. However, among the three drug classes tested and shown to exhibit synergistic activity in whole cell assays, the interaction between 1599 and clarithromycin was the most potent in the ribosomal target assay. Further studies may clarify if strong whole-cell synergism between 1599 and clarithromycin arises from an interaction independent of their known ribosomal binding sites as has been reported for streptogramin ribosome inhibitors^38^.

## Discussion

In the present study, we identified several non-conventional anti-tuberculosis antibiotics that synergized with the anti-tubercular spectinamide 1599 to inhibit growth of *M. tuberculosis*. Hits for whole cell (MIC-based) synergism were validated in clinical isolates, confirming *in vitro* synergism between spectinamides, tetracyclines, macrolides, and closely related lincosamides. The high attrition noted when validating H37Rv hits strongly emphasizes the need to confirm screening hits obtained using a laboratory strain by re-testing with clinical isolates. While *in vitro* synergism occurred at concentrations reportedly achievable *in vivo*, synergism was not strictly recapitulated when combinations were tested in mouse models of acute and chronic tuberculosis infection. Of the three drugs tested for synergism with 1599 *in vivo*, only clarithromycin produced significant additive killing when combined with 1599 in the treatment of an acute tuberculosis infection. This activity was restricted to the 9 day acute infection model, however, as the 1599-clarithromycin combination yielded weaker killing compared to monotherapy with 1599 when tested in the model of chronic tuberculosis infection. This result was unanticipated, and underlines the need for further investigation to better understand the impact of inadequate clarithromycin drug exposure in future combination dosing regimens.

Sufficient concentrations of antibiotic in the bloodstream and affected tissues following dose administration are important for achieving synergism *in vivo*. Indeed, the importance of appropriately matched pharmacokinetic profiles – and the impact of mismatched exposure profiles - has been recently emphasized by the work of Drusano and others^39^. Mismatched exposure profiles can lead to periods of monotherapy, where bacteria are exposed to only the drug with the longer half-life. This can lead to periods of sub-inhibitory concentrations that may permit induction of transient resistance mechanisms and the selection of genetic mutants, as has been reported for the combination of rifampicin and moxifloxacin in *M. tuberculosis*^39^. Indeed we demonstrate here that exposure of *M. tuberculosis* to subinhibitory concentrations of clarithromycin induces clarithromycin resistance and may lead to subsequent loss of synergism with 1599 *in vivo*. As the duration of treatment was extended from dosing BID for 9 consecutive days in the acute infection model to 28 days QD 5 days per week in the chronic infection model employed, it is therefore not surprising to see induction of phenotypic clarithromycin-resistance. The consequence of this was likely induction of transient macrolide resistance, associated loss of clarithromycin efficacy, and subsequent loss of synergism with 1599. Modeling based upon known *in vivo* exposure profiles for the doses used also predicted that increasing dose frequency from once to twice daily and providing treatment 7 days per week should reduce periods in which macrolide resistance is induced in bacilli exposed only to clarithromycin thus enabling synergism *in vivo*. The lack of *in vivo* efficacy produced by these combinations when using a 5/7 day dosing regimen, however, may limit this combination to specific clinical situations where dosing and drug exposures are carefully monitored. Despite unclear efficacy, clarithromycin is included in salvage therapy for MDR tuberculosis when first and second line drugs fail^40^,^41^. Clarithromycin is well tolerated and has an acceptable toxicity profile, making it distinct from most second-line and salvage therapy agents^42^. While sub-inhibitory concentrations of clarithromycin may induce phenotypic macrolide resistance, clarithromycin may offer additional benefits including its anti-inflammatory properties and positive influence on pharmacokinetics of orally co-administered antibiotics, including linezolid for which serum exposure in MDR TB patients increases with clarithromycin^43^, likely through increased oral bioavailability due to inhibition of P-gp mediated efflux. Therefore, future combination trials with clarithromycin should not be necessarily ruled out, but must involve carefully chosen and monitored dosing regimen to ensure optimal exposure. Alternatively, macrolide derivatives that do not induce macrolide resistance may improve prospects for combination treatment with anti-tuberculars, including spectinamides^44^.

The challenge of treating tuberculosis infections is complicated by numerous factors, including *M. tuberculosis*’s impermeable cell wall structure, and its ability to remain latent for decades walled off within granuloma. The complex tissue matrix surrounding granulomas represents a further obstacle that successful anti-tuberculosis must permeate to reach resident bacteria^45^. This challenge is further exacerbated by findings indicating that a single granuloma is composed of several microenvironments and harbors a heterogeneous population of bacilli with distinct metabolic states and drug resistance profiles^5^. Careful design of drug combinations that do not necessarily synergize in the act of killing individual bacilli but rather reach and kill distinct bacterial subpopulations within the infected host, then, may lead to more complete sterilization and successful treatment. Therefore *in vivo* combination trials of 1599 with frontline and developing tuberculosis drugs are of particular interest and ongoing in our laboratories.

## Methods

### Checkerboard synergy assays

Whole cell *in vitro* synergy assays were performed using *M. tuberculosis* strain H37Rv, which was cultured as described previously^21^. In a 96-well assay plate, two-fold serial dilutions of Drug A were prepared in 100 μl of Middlebrook 7H9 media (highest and lowest concentrations in rows A and G, respectively, with no drug in row H). Using a single dip with a 200ss pintool, 0.2 μl of drug B (1599) was transferred to the assay plate columns 1 to 11 of the assay plate, with drug-free DMSO transferred to column 12. To each well of the assay plate 100 μl of mid-log phase bacteria (diluted to OD_600_ of 0.01) was added, and plates incubated for 7 days prior to reading MICs by visual inspection. Fractional inhibitory concentration index (FICI) scores were calculated using the formula [MIC drug B in presence of Drug A]/[MIC of drug B) + [MIC of drug A in the presence of drug B]/[MIC of drug A]. FICI scores were interpreted as follows: synergy (≤0.5), indifference (>0.5–4.0), or antagonism (>4.0)^28^. For each drug combination, FICI ranges were reported from two biologically independent experiments.

### Ethics Statement

*In vivo* efficacy trials were performed at Colorado State University according to Protocol number 13-4263A, approved by the Colorado State University Institutional Animal Care and Use Committee (IACUC).

### *In vivo* dosing selection for combination efficacy trails

Based upon promising *in vitro* synergism, clarithromycin, clindamycin, and doxycycline were chosen for 1599 combination testing in an *in vivo* model of acute *M. tuberculosis* infection^46^. Dosing regimens were selected based on their tolerability and ability to generate therapeutically similar systemic exposures *in vivo* in mice to those reported in the literature for humans. For clindamycin, an oral dose of 100 mg/kg BID was chosen based on mouse PK and plasma protein binding data that indicted free peak plasma concentrations of approximately 4 μg/mL for this dose^47^,^48^. For clarithromycin, an oral dose of 250 mg/kg BID was used, as this dosage was expected to result in free peak plasma concentrations of approximately 1 μg/mL^49^,^50^. For doxycycline, oral administration of 150 mg/kg BID was used, with an expected peak of 0.3 μg/mL for free concentrations^51^,^52^.

### *In vivo* efficacy model of acute tuberculosis infection

Efficacy of antibiotics alone and in combinations was tested as essentially as described previously^35^,^46^,^53^. Briefly, 8 week female GKO mice (C57BL/6-IFNγ knockout from Jackson Laboratories) were infected with a low dose aerosol (LDA; ~100 CFU’s per mouse) of *M. tuberculosis* Erdman, transformed with pFCA-LuxAB. Beginning 13 days post-infection mice were dosed with antibiotics twice daily (BID). Indicated groups of 5 mice per group received monotherapy with 1599 (subcutaneous injection, 150 mg/kg of body weight BID), clarithromycin (oral delivery, 250 mg/kg BID), clindamycin (oral, 100 mg/kg BID), doxycycline (oral delivery, 150 mg/kg BID), or 1599 dosing in combination with clarithromycin, clindamycin, or doxycycline at doses indicated for monotherapy. Mice were dosed for 9 consecutive days. 10 days post-initiation of treatment, lungs were harvested and bacterial loads determined by enumeration of CFU.

### *In vivo* efficacy model of chronic tuberculosis infection

The combination of 1599 with clarithromycin or with clindamycin was evaluated in a chronic infection, using female Balb/c mice (Charles River Labs, Wilmington, MA) infected with a LDA^35^,^54^,^55^,^56^. At 21 days post-infection, mice were treated once daily (QD) for 5 days a week (drug “holiday dosing”). Indicated groups of 6 mice per group received monotherapy with 1599 (subcutaneous injection, 150 mg/kg of body weight QD), clarithromycin (oral delivery, 250 mg/kg QD), clindamycin (oral, 100 mg/kg QD), of 1599 dosing in combination with clarithromycin, or clindamycin, or doxycycline at doses indicated for monotherapy infection model. Lungs were harvested after 28 days of treatment and bacterial loads determined by enumeration of CFU.

### Pharmacokinetic modeling

Plasma concentration-time profiles for 1599 were simulated using a 2-compartment pharmacokinetic model based on pharmacokinetic data after subcutaneous administration in mice^21^. Simulations were performed for 5/7 and 7/7 day dosing with either QD and BID dosing using the software package Phoenix WinNonlin 6.3 (Icon Development Solutions, Hanover, MD).

### Macrolide resistance induction

Checkerboard assays were performed in *M. tuberculosis strains* H37Rv and CDC1551 with and without pre-treatment with 0.1 μg/mL of clarithromycin, a sub-inhibitory concentration that increases the resistance of mycobacteria to clarithromycin.

### Ribosomal inhibition assays

Protein translation assays using mycobacterial ribosomes were performed as described previously modified to measure the inhibition produced by combinations of two compounds^57^. For each drug combination, two-fold dilutions of compound 1599 were combined with two-fold dilutions of partner antibiotic to achieve 35 unique reaction concentration combinations where the concentration of each compound ranged from 1 to 1/16^th^ the previously established ribosomal IC_50_.

## Additional Information

**How to cite this article**: Bruhn, D. F. *et al.* *In vitro* and *in vivo* Evaluation of Synergism between Anti-Tubercular Spectinamides and Non-Classical Tuberculosis Antibiotics. *Sci. Rep.* **5**, 13985; doi: 10.1038/srep13985 (2015).

## Supplementary Material

Supplementary Information

## Figures and Tables

**Table 1:** *In vitro* synergism in *Mycobacterium tuberculosis* laboratory strain H37Rv.

**Drug Class**	**Antibiotic**	**MIC alone**	**MIC in presence of 1599**	**1599 MIC in combination**	**FICI with 1599 (H37Rv)**	**Interaction**	**Number of Replicates**
Acyl-depsipeptide	ADEP4	>20	20	0.6	≤1.0	Indifference	1
Aminocycitol	Spectinomycin	100–200	50–100	0.8	1.0	Indifference	2
Aminoglycoside	Gentamycin	3.1	0.8	0.6	0.75	Indifference	1
Aminoglycoside	Streptomycin	3.1	3.1–6.3	0.8–1.6	2.0–4.0	Indifference	2
Aminoglycoside	Tobramycin	6.3	6.3	1.3	2.0	Indifference	1
Antifolate	Methotrexate (DHFR)	200–>200	100–200	1.3	0.75–≤2.0	Indifference	2
Antifolate	Sulfamethoxazole (DHPS)	200	50	0.8	1.25	Indifference	1
Antifolate	Trimethoprim (DHFR)	>200	12.5–25	0.2–0.3	≤0.13–≤0.19	**Synergism**	3
Antiprotozoal	Chloroquine	>200	>200	2.5	≤3.0	Indifference	1
Cephalosporin	Cefotaxime	>200	>200	1.3	≤2.0	Indifference	1
Carbapenem	Meropenem	6.3	6.3	1.3	≤2.0	Indifference	1
Cyclic polypeptide	Bacitracin	>200	25–50	0.6	≤0.31–≤0.38	**Synergism**	2
Fluoroquinolone	Gatifloxacin	0.2	0.1	0.6	1.0	Indifference	1
Glycopeptide	Vancomycin	50	3.1	0.3–0.6	0.19–0.31	**Synergism**	2
Glycycline	Tigecycline	6.3	0.8	0.6	0.6	Indifference	1
Lincosamide	Clindamycin	>200–>400	0.1–6.3	0.02–0.2	≤0.02–≤0.16	**Synergism**	3
Lincosamide	Lincomycin	>200	1.6–12.5	0.04–0.2	≤0.07–≤0.16	**Synergism**	2
Lincosamide	Pirlimycin	>200	25–50	0.3	≤0.31–≤0.38	**Synergism**	2
Macrolide	Azithromycin	>400–>800	6.3–12.5	0.3–0.4	≤0.13–≤0.28	**Synergism**	3
Macrolide	Clarithromycin	12.5–48	0.1–5.6	0.04–0.05	0.07–0.35	**Synergism**	3
Macrolide	Erythromycin	>200–800	12.5–50	0.2–0.4	0.16–≤0.28	**Synergism**	3
Nitroimidazole	Metronidazole	>200	200	0.6	≤1.0	Indifference	1
Nitroimidazole	Nitrofurantoin	50	25	0.6	1.0	Indifference	1
Oxazolidinone	Linezolid	3.1	3.1	0.1	1.5	Indifference	1
Tetracycline	Doxycycline	6.3–50	0.4–0.8	0.1–0.3	0.08–0.31	**Synergism**	5
Tetracycline	Minocycline	25	1.6	0.1	0.13	**Synergism**	1
Tetracycline	Tetracycline	25–50	0.8–1.6	0.1–0.2	0.08–0.13	**Synergism**	2

Whole cell, checkerboard assays were used to characterize interaction of 1599 with indicated antibiotics. Assays were performed with *Mycobacterium tuberculosis* strain H37Rv. Fractional Inhibitory Concentration Index (FICI) scores were interpreted as follows: synergy (≤0.5), indifference (>0.5–4.0) or antagonism (>4.0) and MICs are in μg/mL. Results are presented as the range from the indicated number of biologically independent experimental replicates. A less than or equal to symbol (≤) preceding a FICI score indicates that an MIC was higher than the greatest concentration tested, which was used in FICI calculation.

**Table 2:** Validation of Preliminary Hits.

**Antibiotic**	**FICI Score with 1599**				**FICI Score with other Spectinamides and Spectinamides (strain H37Rv)**			**Consensus Interaction**
	**H37Rv**	**Erdman**	**TN14149**	**TN043**	**1329**	**1445**	**Spectinomycin**	
Bacitracin	≤0.31–≤0.38	≤1.06^*^	≤1.0^*^	≤0.75^*^	nd	nd	nd	Indifference
Clarithromycin	0.12–0.34	0.06–0.07	0.09–0.15	0.02^*^	0.003–0.02	0.03^*^	0.04–0.37	**Synergism**
Clindamycin	≤0.02–≤0.07	≤0.03^*^	≤0.05^*^	≤0.05^*^	≤0.03–≤0.06	≤0.03–≤0.06	≤0.13–≤0.5	**Synergism**
Doxycycline	0.08–0.31	0.09	0.5^*^	0.25^*^	0.02^*^	0.02^*^	0.03–0.31	**Synergism**
Tetracycline	nd	nd	nd	nd	0.01^*^	0.02^*^	0.02	**Synergism**
Trimethoprim	≤0.13–0.19	≤0.13^*^	≤2.0^*^	≤0.5^*^	≤0.75–≤2.0	≤0.75–≤2.0	nd	Indifference
Vancomycin	nd	nd	nd	nd	1.0–1.5	1.0	1.0	Indifference

Whole cell, checkerboard assays were used to characterize interaction of spectinamides (1599, 1329, 1445) or parent spectinomycin with indicated antibiotics. Assays were performed with *Mycobacterium tuberculosis* laboratory strains (H37Rv, Erdman) or clinical isolates (TN14149, TN043), as indicated.

Fractional Inhibitory Concentration Index (FICI) scores were interpreted as follows: synergy (≤0.5), indifference (>0.5–4.0) or antagonism (>4.0) and are representative of 2–3 independent experiments, except where *indicates results are from a single experiment or “nd” indicates not determined.

**Table 3:** Reduction in MIC produced by sub-inhibitory concentrations of compound 1599.

**Antibiotic**	**MIC (μg/mL)**		**% MIC**
	**(−) 1599**	**(+) 1599**	
Clarithromycin	25–50	0.1	0.2–0.4%
Clindamycin	>200	6–25	<1.5–6.3%
Doxycycline	6.3–50	0.4–1.6	3.2–6.5%
Tetracycline	50	6.3	12.6%

MICs of macrolides and tetracyclines in the absence (−) or presence (+) of 0.6 μg/mL of spectinamide 1599. The % of original MIC values were calculated by dividing the MIC in the presence of 1599 by the MIC in the absence of 1599. Results presented are from two biologically independent experiments with *Mycobacterium tuberculosis* strain H37Rv.
