# Supplementary material for: In vitro and in vivo Evaluation of Synergism between Anti-Tubercular Spectinamides and Non-Classical Tuberculosis Antibiotics
Source: Sci Rep. 2015 Sep 14;5:13985. doi: 10.1038/srep13985 (PMC4568539; doi:10.1038/srep13985)
Supplement: Supplementary Information [file srep13985-s1.doc]

***In vitro* and *in vivo* Evaluation of Synergism between Anti-Tuberculosis Spectinamides and Non-Classical Tuberculosis Antibiotics**.

David F. Bruhn,^1^ Michael Scherman,^2^ Jiuyu Liu,^1^ Dimitri Scherbakov,^3^ Erik C. Böttger,^3^ Bernd Meibohm,^4^ Anne J. Lenaerts,^2^  & Richard E. Lee,^1^

1. Department of Chemical Biology and Therapeutics, St. Jude Children's Research Hospital, Memphis, Tennessee, USA

2. Mycobacterial Research Laboratories, Department of Microbiology, Colorado State University, Fort Collins, Colorado, USA

3. Institut für Medizinische Mikrobiologie, Nationales Zentrum für Mykobakterien, Universität Zürich, Zürich, Switzerland

4. Department of Pharmaceutical Sciences, College of Pharmacy, University of Tennessee Health Science Center, Memphis, TN, USA

**Contents**

**Table S1:** Statistical analysis of lung data for murine acute infection model……………..page S2

**Table S2:** Statistical analysis of lung data for murine chronic infection model…………..page S3

**Table S3:** Impact of pre-treatment with a fixed sub-inhibitory concentration of clarithromycin on *in vitro* synergism against strain H37Rv…....………………………………….…………..page S4

**Table S4:** Impact of pre-treatment with a fixed sub-inhibitory concentration of clarithromycin on *in vitro* synergism against strain CDC1551 …...………………………………………......page S4

**Figure S1:** Protein synthesis inhibition by two-dimensional compound titration…………page S5

**­­­Supplementary Table S1:** Statistical analysis of lung data for murine acute infection model

| **Treatment Group Comparison** | **Difference of Means (Log10CFU)** | **P Value** | **Statistically Significant?** | **Interaction** |
| --- | --- | --- | --- | --- |
| Carrier vs. 1599  (150 mg/kg BID) | 1.856 | <0.001 | **Yes** | - |
| Carrier vs. Clarithromycin  (250 mg/kg BID) | ­­1.729 | <0.001 | **Yes** | - |
| Carrier vs. Clindamycin  (100 mg/kg BID) | 0.529 | 0.319 | No | - |
| Carrier vs. Doxycycline  ­­(150 mg/kg BID) | 0.816 | 0.012 | **Yes** | **-** |
| 1599 vs. 1599/Clarithromycin | 0.896 | 0.008 | **Yes** | **Additivity** |
| 1599 vs. 1599/Clindamycin | 0.293 | 0.967 | Do Not Test | Indifference |
| 1599 vs.1599/Doxycycline | 0.351 | 0.894 | Do Not Test | Indifference |

CFU in indicated organ were enumerated by plating serial dilutions of organ homogenate onto drug-free 7H11 agar plates. Difference of mean (Log_10_) was calculated by subtracting bacterial titer of second indicated group from that of the first.

Statistical significance was calculated by Tukeys’s multiple comparison test.

**Supplementary Table S2:** Statistical analysis of lung data for murine chronic infection model

| **Treatment Group Comparison** | **Difference of Means (Log10CFU)** | **P Value** | **Statistically Significant?** | **Interaction** |
| --- | --- | --- | --- | --- |
| Initiation of Treatment vs Carrier | 0.596 | <0.001 | **Yes** | **-** |
| Carrier vs. 1599  (150 mg/kg QD) | 1.192 | <0.001 | **Yes** | **-** |
| ­­Carrier vs. Clarithromycin  (250 mg/kg QD) | 0.0116 | 1.000 | Do Not Test | - |
| Carrier vs. Clindamycin  (100 mg/kg QD) | 0.113 | 0.978 | Do Not Test | - |
| 1599 vs. 1599/Clarithromycin | -0.429 | <0.001 | **Yes** | **Potential Antagonism** |
| 1599 vs. 1599/Clindamycin | =0.346 | 0.013 | **Yes** | **Potential Antagonism** |

CFU in indicated organ were enumerated by plating serial dilutions of organ homogenate onto drug-free 7H11 agar plates. Difference of mean (Log_10_) was calculated by subtracting bacterial titer of second indicated ­group from that of the first.

Statistical significance was calculated by Tukeys’s multiple comparison test.

**Supplementary Table S3:** Impact of pre-treatment with a fixed sub-inhibitory concentration of clarithromycin on *in vitro* synergism against strain H37Rv

| **Group** | **H37Rv** | | | | | |
| --- | --- | --- | --- | --- | --- | --- |
|  | **Clarithromycin** | | **1599** | | **FICI** | **Lowest Conc. of Clar. To reduce 1599 MIC** |
|  | **MIC alone** | **MIC in combo.** | **MIC alone** | **MIC in combo.** |  |  |
| Untreated | 50 - >50 | 0.01- 0.1 | 2.5 | 0.2 - 0.3 | 0.06- 0.13 | 0.1-0.8 |
| Pre-treated | >50 - >200 | 25-50 | 1.3 | 0.6 - 0.6 | 0.6 -1.0 | 200 - >200 |

Checkerboard assays were performed in *M. tuberculosis* strain H37Rv with and without pre-treatment with 0.1 µg/mL of clarithromycin, a sub-inhibitory concentration that increases the resistance of mycobacterium to clarithromycin. The lowest concentration of clarithromycin (Clar.) required to reduce 1599 MIC 4 fold is noted in µg/mL.
Results from 2 biologically independent experiments are presented as a range, with MIC values expressed in µg/mL.

**Supplementary Table S4: Impact of pre-treatment with a fixed sub-inhibitory concentration of clarithromycin on *in vitro* synergism against strain CDC1551**

| **Group** | **CDC1551** | | | | | |
| --- | --- | --- | --- | --- | --- | --- |
|  | **Clarithromycin** | | **1599** | | **FICI** | **Lowest Concentration of Clarithromycin Required to Reduce 1599 MIC** |
|  | **MIC alone** | **MIC in combo.** | **MIC alone** | **MIC in combo.** |  |  |
| Untreated | 25 | 0.02 - 0.1 | 1.3 | 0.1- 0.01 | 0.01- 0.06 | 0.4 -0.8 |
| Pre-treated | 200 - >200 | 0.2 - 3.1 | 1.3 | 0.3 - 1.3 | 0.25 - 0.26 | 50- 200 |

Checkerboard assays were performed in *M. tuberculosis* strain CDC1551 with and without pre-treatment with 0.1 µg/mL of clarithromycin, a sub-inhibitory concentration that increases the resistance of mycobacterium to clarithromycin. The lowest concentration of clarithromycin (Clar.) required to reduce 1599 MIC 4 fold is noted in µg/mL.
Results from 2 biologically independent experiments are presented as a range, with MIC values expressed in µg/mL.

**Supplementary Figure S1: Protein synthesis inhibition by two-dimensional compound titration**

|  |  | **Lee1599 (µg/mL)** | | | | | |
| --- | --- | --- | --- | --- | --- | --- | --- |
|  |  | 0.00 | 0.08 | 0.16 | 0.31 | 0.62 | 1.24 |
| **Clarithromycin (µg/mL)** | 0.00 | 0 | 10 | 18 | 23 | 35 | 46 |
|  | 0.06 | 2 | 10 | 20 | 27 | 34 | 35 |
|  | 0.12 | 11 | 19 | 25 | 32 | 36 | 48 |
|  | 0.24 | 16 | 32 | 36 | 36 | 45 | 53 |
|  | 0.48 | 39 | 42 | 51 | 53 | 57 | 60 |
|  | 0.96 | 64 | 65 | 62 | 64 | 70 | 68 |
|  |  |  |  |  |  |  |  |
|  |  | **Lee1599 (µg/mL)** | | | | | |
|  |  | 0.00 | 0.08 | 0.16 | 0.31 | 0.62 | 1.24 |
| **Clindamycin (µg/mL)** | 0.00 | 0 | 5 | 14 | 20 | 35 | 37 |
|  | 1.49 | 14 | 22 | 20 | 29 | 43 | 38 |
|  | 2.98 | 19 | 16 | 24 | 33 | 37 | 41 |
|  | 5.96 | 27 | 26 | 34 | 38 | 42 | 46 |
|  | 11.92 | 34 | 37 | 42 | 39 | 49 | 51 |
|  | 23.83 | 48 | 44 | 48 | 48 | 52 | 59 |
|  |  |  |  |  |  |  |  |
|  |  | **Lee1599 (µg/mL)** | | | | | |
| **Tetracycline (µg/mL)** |  | 0.00 | 0.08 | 0.16 | 0.31 | 0.62 | 1.24 |
|  | 0.00 | 0 | 12 | 18 | 28 | 37 | 45 |
|  | 0.15 | 12 | 16 | 21 | 24 | 32 | 42 |
|  | 0.30 | 18 | 22 | 27 | 30 | 41 | 44 |
|  | 0.60 | 21 | 28 | 35 | 37 | 46 | 50 |
|  | 1.21 | 35 | 41 | 46 | 50 | 53 | 57 |
|  | 2.41 | 48 | 52 | 53 | 59 | 59 | 66 |

Translation assays were carried out using the indicated concentration of compounds indicated. Compound concentrations are indicated (in μg/mL) outside of boxes indicating the percent inhibition relative to untreated control group. Reaction conditions yielding a greater than 50% inhibition in protein synthesis are indicated in red.
